# Comparison of harmonic blade versus traditional approach in canine patients undergoing spinal decompressive surgery for naturally occurring thoracolumbar disk extrusion

**DOI:** 10.1371/journal.pone.0172822

**Published:** 2017-03-02

**Authors:** Bianca F. Hettlich, Laurie Cook, Cheryl London, Geoffrey T. Fosgate

**Affiliations:** 1 Department of Veterinary Clinical Sciences, College of Veterinary Medicine, Ohio State University, Columbus, Ohio, United States of America; 2 Department of Production Animal Studies, Faculty of Veterinary Science, University of Pretoria, Onderstepoort, South Africa; University of Bari, ITALY

## Abstract

**Objectives:**

To assess feasibility of the harmonic Osteovue blade (HOB) for use in the soft tissue approach for dogs undergoing hemilaminectomy and to compare outcomes between dogs undergoing HOB or traditional approach (TRAD).

**Methods:**

A prospective randomized clinical trial was performed using 20 client-owned dogs with thoracolumbar intervertebral disk extrusion requiring hemilaminectomy. Dogs were randomly assigned to HOB or TRAD. Neurologic function and pain scores were assessed pre-operatively. Intraoperative blood loss and surgical approach time as well as postoperative pain and wound healing scores were recorded. Additionally, neurologic recovery and owner perceived quality of life were recorded at day 10 and 30 postoperative.

**Results:**

There was no significant difference in sex distribution, weight, age, preoperative neurological grade and pain score, and perioperative outcome measures between groups. Intraoperative total blood loss was minimal for HOB and TRAD (median: 0 ml (range 0–9) and 2.2 ml (range 0–6.8), respectively; p = 0.165) and approach times were similar (median: 7 min (range 5–12) and 8 min (range 5–13), respectively; p = 0.315). While changes in wound healing scores were similar, changes in postoperative pain scores and neurological function were significantly improved in the HOB compared to the TRAD group. Postoperative complications in the HOB group consisted of automutilation of part of the incision and development of a small soft, non-painful subcutaneous swelling in 1 dog each.

**Conclusions:**

The HOB is a safe and effective tool for the soft tissue approach for routine spinal surgery in dogs and is associated with decreased pain and increased neurological function post-surgery.

## Introduction

Laminectomies are among the most common procedures performed in canine neurosurgery. Soft tissue dissection during the surgical approach is similar to that used in human spine surgery, traditionally incorporating a mixture of sharp dissection with scalpel blade or scissors, blunt dissection with periosteal elevators, and electrocautery [1] While bleeding during the approach to the canine spine is rarely severe enough to require blood transfusions, even small bleeds can slow down surgery and impair visualization during subsequent decompression, potentially impacting clinical outcomes. In human spine surgery, bleeding can be significant and often requires either allo- or autogeneic blood transfusions [2, 3]. Therefore, reduction of intraoperative bleeding is of clinical importance for the patient to decrease transfusion requirements.

Various methods including acute normovolemic hemodilution, controlled hypotensive anesthesia, or medical intervention with drugs such as anti-fibrinolytics and vasoconstrictors have been reported to decrease intraoperative bleeding during human spine surgery [4–10]. Alternatively, vascular coagulation using electrocautery or ultrasonic coagulation can be used. Ultrasonic technology is based on cutting and coagulation of tissues through mechanical dissection rather than electrical current, thereby avoiding charring, development of smoke, resulting in decreased thermal injury. [11–14]. Experimental studies have shown that dissection using ultrasonic surgical blades causes less damage to nearby neurovascular structures and decreases acute inflammation while improving early neovascularization in wound healing compared to electrosurgery [15–18]. Clinical studies on a variety of human soft tissue and orthopedic surgical procedures also support the efficacy of ultrasonic surgical blades to reduce surgical time and decrease blood loss compared to electrocautery [19–24].

Standard instruments for soft tissue approach to the human spine include electrocautery and periosteal elevators such as the Cobb elevator. To circumvent some of the potential complications associated with these standard instruments, the harmonic scalpel was developed. An earlier model of the harmonic scalpel (Ultracision; Ethicon Endo-Surgery, Cincinnati, OH) was previously evaluated for its potential efficacy in posterior spinal instrumentation in humans [25]. In this study, use of the Ultracision led to significantly decreased surgical time and lower blood loss. Advances in harmonic blade development have led to a more robust, spade-like blade (Osteovue; Ethicon Endo-Surgery, Cincinnati, OH), which is able to elevate tissue off bone, spot coagulate vessels and dissect through tissues. As such, the novel harmonic Osteovue blade allows the use of a single instrument to elevate and cut paraspinous soft tissues and to simultaneously coagulate to achieve hemostasis.

The purpose of this study was to establish feasibility and safety of the harmonic Osteovue blade (HOB) in a relevant spontaneous large animal model of disease: canine patients with spontaneous thoracolumbar (TL) intervertebral disk extrusion (IVDE) requiring spinal cord decompression via hemilaminectomy. Dogs undergoing surgery for IVDE were randomized to have HOB or traditional sharp dissection and electrocautery (TRAD) used for the surgical approach. Outcome assessments included surgical approach time, degree of intraoperative bleeding, postoperative surgical pain scores and wound healing scores. Results of this trial demonstrated that the HOB was safe and effective for the soft tissue approach for routine spinal surgery in dogs and was associated with decreased pain and increased neurological function post-surgery.

## Methods

### Study design

The clinical study was conducted as a blinded, randomized clinical trial and was coordinated by the Clinical Trials Office at the OSU CVM following Good Clinical Practice guidelines [26]. This clinical study was approved by the Clinical Research Advisory Committee at the OSU CVM, the primary committee that regulates and oversees research in client owned animals at the OSU CVM Veterinary Medical Center. Per policy established by the IACUC at OSU, the study received a waiver from the need for full approval as the scope of research was not deemed to go beyond the standard of care for treating dogs with IVDE (i.e. decompressive surgery is treatment of choice) and similar HOB devices have already been approved by the FDA. As such, the welfare and care of the dogs enrolled in this study was monitored by the Clinical Research Advisory Committee.

Dogs with naturally occurring IVDE involving the TL spine undergoing decompressive surgery by hemilaminectomy were eligible for inclusion. Dogs had to meet the following criteria: intact deep nociception in pelvic limbs on pre-operative neurological evaluation, evidence of spinal cord compression secondary to a single site IVDE between T10 and L6 based on advanced imaging, body weight between 4–30 kg, and lack of significant underlying systemic disorders including cardiac, endocrine, renal or hepatic disease that may impair postoperative recovery and wound healing. Informed owner consent was required prior to inclusion of dogs into the study. All dogs underwent neurological examination and were graded according to the following scale: 0 = normal, 1 = spinal hyperesthesia only, 2 = ambulatory paraparesis, 3 = non-ambulatory paraparesis, 4 = paraplegia, deep nociception present.

Following enrollment, dogs were randomly assigned to undergo surgery using the traditional approach with sharp dissection and electrocautery (TRAD; n = 10) or an approach using the Harmonic Osteovue Blade (HOB; n = 10). Owners were blinded to the type of approach used on their dog. Diagnostic imaging included computed tomography (CT), CT plus intrathecal contrast or magnetic resonance imaging (MRI). For all dogs, except for 2 that were younger than 3 years of age, complete blood counts and chemistry panels were performed. Pre- and immediate postoperative PCV /TP were recorded for all dogs. The anesthetic regimen was routine for all patients and included the following: acepromazine (0.02–0.05mg/kg IM or IV) and hydromorphone (0.05–0.1mg/kg IM or IV) for premedication; propofol (4-6mg/kg IV) for induction; and isoflurane gas inhalation for maintenance anesthesia. Intraoperatively, dogs received constant rate infusions of lactated ringer solution (3-5mg/kg/hr IV) and fentanyl (5-10mcg/kg/hr IV)If required for cardiovascular support, dogs received glycopyrrolate (5mcg/kg IV). All dogs received cefazolin perioperatively (22mg/kg IV every 90 minutes).

### Surgical procedures

All dogs underwent hemilaminectomy via a standard dorsolateral approach [27]. Surgical time was recorded separately for approach (skin incision to complete exposure for hemilaminectomy with hemorrhage controlled), hemilaminectomy (end of approach until completion of decompressing and final lavage), and closure (final lavage to completion of skin closure). To maintain uniformity in technique, approach and closure of all dogs were performed one of two individuals during the study: B. Hettlich (Diplomate of the American College of Veterinary Surgeons) or L. Cook (Diplomate of the American College of Veterinary Internal Medicine, Specialty of Neurology). Following completion of the surgical approach, the hemilaminectomies were performed by either Hettlich or Cook with assistance from surgical or neurology residents at the OSU CVM. The skin incision was performed using a scalpel blade for all dogs. All other soft tissue dissection thereafter was performed using either the TRAD or HOB approach.

**Fig 1:**
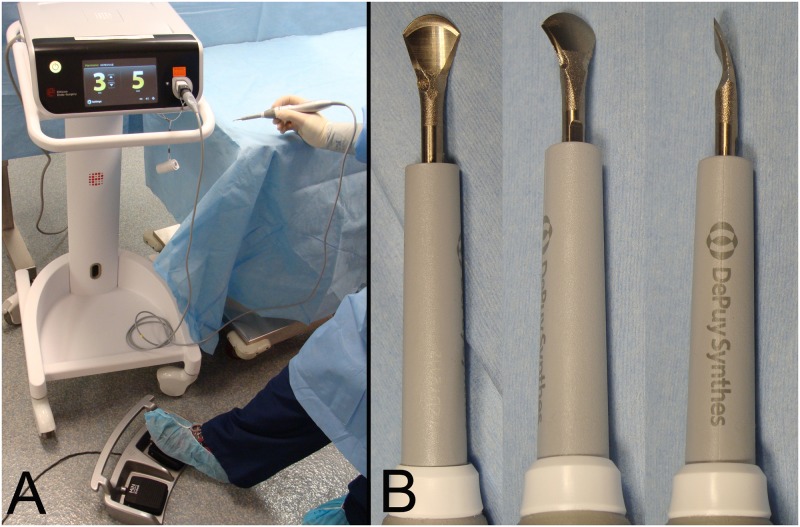
Harmonic Osteovue blade set-up. A: photographs of the generator, foot switch and hand piece (Ethicon Endo-Surgery, Inc.). B: photographs of the harmonic Osteovue blade (Ethicon Endo-Surgery, Inc.) showing the spade-like and curved shape of the blade tip.

In the TRAD approach, scalpel blade, tenotomy scissors, periosteal elevators and electrocautery were used for sharp and blunt dissection to elevated paraspinous musculature, and hemostasis was achieved with electrocautery (Bovie Medical Corporation, Clearwater, FL). In the HOB approach, all dissection was performed with the harmonic Osteovue blade, using a blunt periosteal elevator for retraction only. The HOB setup included a hand piece, generator, foot switch and the Osteovue blade itself (Fig 1).

Throughout the procedure, the maximum setting for cutting was used where the HOB moves at 55,000 Hz with an amplitude of 70 micro meters. Hemorrhages were controlled with the harmonic blade only. In both groups, final exposure for hemilaminectomy was provided using Gelpi retractors.

During the approach, no wound lavage was performed and no sponges were used. Blood was cleared from the surgical field using suction only. At the end of the approach (vertebral exposure sufficient for hemilaminectomy, bleeding controlled), blood was collected from the suction container and the amount measured to determine blood loss. Observations were made regarding amount of tissue bleeding during the approaches and subjective hemorrhage control by cautery and HOB.

Closure was the same for all dogs using either 2.0 or 3.0 polydioxanone (PDS; Ethicon Inc., Somerville, NJ) in a continuous pattern for fascia, 3.0 poliglecaprone 25 (Monocryl, Ethicon Inc., Somerville, NJ) in a continuous patter for a combined closure of the subcutaneous and intradermal layers, and 316L stainless steel staples for skin closure (Covidien llc, Mansfield, MA). A Telfa island dressing was applied to the incision until the next day (Kendall Healthcare Prod., Mansfield MA).

After completion of surgery, all dogs received a transdermal fentanyl patch (2-4mcg/kg/hr) and recovered on IV fluids and a constant rate infusion (CRI) of Fentanyl for a minimum of 12 hours. If preoperative anti-inflammatory drugs had been administered, they were continued as necessary. If no preoperative drugs had been administered, dogs received postoperative oral NSAIDs. Additionally, dogs received oral tramadol as needed after the Fentanyl CRI was discontinued. Some dogs also received oral gabapentin for additional pain management.

### Postoperative clinical evaluation

For the duration of hospitalization, each dog underwent daily neurological assessment. Additionally, wound related parameters and level of pain were evaluated daily by two clinicians independently, one of which was unaware of the type of surgical approach. The wound assessment form included questions using numeric rating related to discharge, swelling, bruising and incisional pain (S1 File). Wound assessment was based on visual scoring using a non-validated scoring system. Scores could range from 0 to 15 with higher values indicative of increasing wound healing complications. Photographs of the incision were obtained at each evaluation (S1 Fig). These served as a placeholder and were not used as the basis for assessment of wound healing as they represented a single point of reference rather than the assessment of the investigator. The pain assessment form included a visual analog scale (VAS) as well as questions from the Glasgow composite pain scale for dogs [28] (S2 File). Pain values from the questionnaire could range from 0 to 16 with lower values indicating lower pain. Pain on the VAS could range from 0 to 10 with 0 being no pain. Pain and wound healing scores were calculated for each assessment period and the average value was generated from the 2 observers.

Following discharge from the hospital, dogs were evaluated at the OSU VMC at 2 additional time points: 10 days postoperatively at time of skin staple removal and 30 days postoperatively at time of neurologic recheck. At both time points, a complete neurological examination was performed and the current neurologic grade was recorded. This was used to determine neurologic improvement between the preoperative neurologic grade and the subsequent evaluation time points (change in neurologic grade). Two independent evaluators assessed pain and wound healing as described previously. Improvement of pain was also determined relative to the preoperative values and the subsequent evaluation time points and calculated by the change in pain scores and pain VAS.

### Owner questionnaire

Owners completed a quality of life (QOL) questionnaire form prior to the surgical procedure and at days 10 and 30 post surgery [29]. This previously published QOL questionnaire was changed slightly by adding 3 wound related questions and modifying the mobility related questions to account for neurological deficits (S3 File). Questions were answered on a Likert scale from 1 to 5 with 1 indicating strong disagreement and 5 strong agreement. For analysis, the scale for negative questions was reversed so that better outcomes had higher scores. The combined QOL questionnaire score could range from 1 to 5 with higher values indicating a better owner perceived quality of life. Additionally, owners assessed their dog’s QOL on a VAS. Following discharge from the hospital, owners were asked to complete a daily patient diary to record swelling, discharge or pain associated with the surgical wound.

### Statistical analysis

Quantitative data were assessed for normality by plotting histograms, calculating descriptive statistics, and performing the Anderson-Darling test (MINITAB Statistical Software, Release 13.32, Minitab Inc, State College, Pennsylvania, USA). Quantitative data were described using the median and range due to the small sample size and the apparent violation of the normality assumption. Comparisons between the Harmonic Osteovue Blade (HOB) and traditional surgery approach (TRAD) groups were performed using Mann-Whitney U tests (IBM SPSS Statistics Version 22, International Business Machines Corp., Armonk, NY, USA). Categorical data were described using proportions and 95% mid-P exact confidence intervals. Categorical data were compared between HOB and TRAD surgical approaches using chi-square and Fisher exact tests (Epi Info, version 6.04, CDC, Atlanta, GA). Statistical analyses for the subjective scales (the ordinal pain and incision healing scales) were performed on the average value of two independent evaluators and inter-observer repeatability was assessed by calculating the coefficient of variation. Non-parametric Mann-Whitney U tests were used to compare changes in the ordinal scale data between HOB and TRAD groups. Statistical results were interpreted at the 5% level of significance.

## Results

### Patient demographics and clinical presentation

Dogs enrolled into this study included 9 Dachshunds, 6 mixed breed dogs, 2 Beagles, 1 Wheaton Terrier, 1 Silky Terrier and 1 Shih Tzu. Median weight was 9.4kg (range: 4–29.1kg). Median age was 75.8 months (range: 30–144 months). There were 10 spayed females, 7 neutered males, 2 intact females, and 1 intact male dog. There was no significant difference of sex distribution between the 2 groups (p = 0.170). Pre-referral treatments included administration of non-steroidal anti-inflammatory drugs (NSAIDs), corticosteroids, tramadol, gabapentin, and cephalexin. 4 dogs received no medical treatment prior to referral. Preoperative blood work in 18 dogs did not reveal evidence of underlying systemic disorders. Complete blood work was not performed in 2 dogs less than 3-years of age that had no history or evidence of systemic disease on physical examination. Preoperative factors were not significantly different between surgical groups (Table 1).

**Table 1:** Comparison of various *preoperative* factors between dogs undergoing thoracolumbar spinal decompressive surgery for intervertebral disk extrusion selected for Harmonic Osteovue Blade (HOB; n = 10) versus traditional surgical approach (TRAD; n = 10).

Variable	HOB		TRAD		P value*
	Median	Range	Median	Range	
Weight (kg)	8.7	4.1, 29.1	6.8	4.0, 18.2	0.393
Age (months)	80	30, 118	68	53, 144	0.684
Neurologic grade	3.5	2, 4	2.5	1, 3	0.075
Pain score	6	1, 9	3.5	2, 7	0.052
Pain VAS	3.4	1.0, 7.5	2.0	0.7, 4.5	0.079
QOL (questionnaire)	3.3	2.7, 4.4	2.8	2.5, 4.1	0.200
QOL VAS	2.6	2.1, 3.7	1.4	0, 7.5	0.570
Owner perceived function	4	2, 4	4	1, 4	0.481

QOL = quality of life.

VAS = visual analog scale. QOL = quality of life.

* Based on chi-square or Fisher exact tests for categorical data and Mann-Whitney U tests for quantitative data comparing dogs with HOB and TRAD.

Neurologic grades measured preoperatively were: grade 1 (n = 1), grade 2 (n = 7), grade 3 (n = 7), and grade 4 (n = 5). Advanced imaging consisted of CT (n = 15), CT/myelogram (n = 4), and MRI (n = 1) and localized a single site extradural compressive lesion to the following locations: T11-T12 (n = 3), T12-T13 (n = 2), T13-L1 (n = 4), L1-L2 (n = 3), L2-L3 (n = 3), L3-L4 (n = 4), and L4-L5 (n = 1).

### Surgical procedure

Perioperative factors were not different between surgical groups (Table 2).

**Table 2:** Quantitative comparisons of various *perioperative* factors between dogs undergoing thoracolumbar spinal decompressive surgery for intervertebral disk extrusion using either Harmonic Osteovue Blade (HOB; n = 10) or traditional surgical approach (TRAD; n = 10).

Variable	HOB		TRAD		P value*
	Median	IQR	Median	IQR	
Approach length (mm)	56	40, 90	60	45, 70	0.280
Duration (min)					
Approach	7	5, 12	8	5, 13	0.315
Hemilaminectomy	47	35, 63	42	20, 62	0.165
Closure	8.5	8, 15	10	6, 15	0.853
Total	65	48, 80	58	36, 79	0.353
Blood loss					
Total (ml)	0	0, 9	2.2	0, 6.8	0.165
ml per kg	0	0, 0.9	0.4	0, 1.1	0.063
PCV (%)					
Pre-operative	52	45, 61	50	45, 55	0.579
Post-operative	39	32, 44	40	34, 44	0.408
Change in PCV	16	1, 22	9	1, 17	0.122
Total protein (mg/dl)					
Pre-operative	7.1	6.0, 8.2	7.2	6.3, 8.0	0.796
Post-operative	6.2	5.6, 7.0	6.0	5.7, 6.6	0.696
Change in TP	1.1	-1, 1.6	1.0	0.3, 2.0	0.897
Hospitalization (days)	2	2, 3	2	2, 5	0.631

IQR = interquartile range.

*Based on Mann-Whitney U tests.

Bleeding from soft tissues was subjectively controlled by HOB and TRAD and blood loss during the approach in both groups was considered minimal. Neither HOB nor electrocautery was used for venous sinus bleeding from within the vertebral canal. Venous sinus bleeding did not occur or was considered minor in 19 of 20 dogs. In 1 dog (TRAD group), venous sinus bleeding was considered moderate and part of an absorbable gelatin sponge was used to aid coagulation. The post-operative PCV/TP was lower in all dogs compared to pre-operative; however, there was no difference between the 2 treatment groups.

There was one intraoperative complication in the HOB group. The spinous process of one of the approached vertebrae was found to be fractured at the conclusion of the hemilaminectomy and was subsequently removed. No obvious bone damage in this area had been noted prior to hemilaminectomy. There were no intraoperative complications noted in the TRAD group.

### Postoperative clinical evaluation

Improvement over time in neurological grade and pain was documented as the change in grade/score compared to initial preoperative values. The HOB had a better improvement in neurological grade at time of discharge and at day 10 recheck. At discharge, neurologic function was grade 1 (n = 1), grade 2 (n = 7), grade 3 (n = 7), and grade 4 (n = 4). By day 10, all but one dog had improved in neurologic function and all had motor function present (grade 2, n = 12; grade 3, n = 7; grade 4, n = 1). By day 30, 2 dogs were normal (grade 0) and the remaining 17 were able to walk well with residual ataxia and some weakness (grade 2).

The postoperative wound healing scores were not significantly different between the 2 groups at any time point. These scores were solely based on visual scoring of the wound and not on photographic appearance. Postoperative wound complications occurred in 2 HOB dogs. They consisted of formation of a small, non-painful swelling associated with the incision noted at day 30 recheck (suspected seroma, treated conservatively) and self-mutilation of an apparently healed incision with partial wound opening on day 10 without prior wound related issues. The latter underwent primary closure of the cleaned wound and demonstrated routine healing thereafter. There were no postoperative complications noted in the TRAD group.

The HOB had a better improvement in postoperative pain (both questionnaire scores and VAS) over the entire study period. Two dogs experienced spinal pain presumably unrelated to the study/type of approach. One dog in the HOB group was described by the owner as being pain free until sudden onset back pain occurred on day 10 (the day of evaluation). Pain resolved in this dog within a few days with conservative management; no further diagnostics were pursued. One dog in the TRAD group continued to demonstrate general back pain and pain on palpation of the surgery site after discharge from the hospital. Back pain had deteriorated significantly at the 10 day evaluation and the dog was euthanized on day 14 due to lack of response to medical therapy. This dog had demonstrated normal wound healing without incisional complications up to this point. No further diagnostics were pursued prior to euthanasia. Results of comparisons of postoperative factors are listed in Table 3. Unlike incision healing scores, neurologic grades and pain scores/VAS started with different preoperative baseline values, the improvement was therefore reflected as a change in grades and scores compared to preoperative values. Values at each individual time point can be found in S1 Data.

**Table 3:** Qualitative comparisons of various *postoperative* factors between dogs undergoing thoracolumbar spinal decompressive surgery for intervertebral disk extrusion using either Harmonic Osteovue Blade (HOB; n = 10) or traditional surgical approach (TRAD; n = 10).

Variable	HOB		TRAD		P value*
	Median (n)	IQR	Median (n)	IQR	
Change in neurologic grade relative to preop					
At discharge	1	-1, 2	0	-1, 1	0.029
10 day follow-up	1.5	0, 3	0	-1, 1	0.029
30 day follow-up	2	0, 4	1	0, 1	0.079
Change in pain scores relative to preop					
At discharge	6.0	0.5, 8.0	2.8	1.0, 6.0	0.029
10 day follow-up	4.5	0, 8.5	2.3	0.5, 7.0	0.089
30 day follow-up	6.0	1.0, 8.0	3.0	1.5, 6.0	0.017
Change in pain VAS relative to preop					
At discharge	3.2	1.0, 7.1	1.7	-0.8, 4.0	0.043
10 day follow-up	3.3	1.0, 7.0	1.6	-0.1, 2.4	0.007
30 day follow-up	3.3	1.0, 7.4	1.7	0, 2.5	0.013
Incision healing score					
Day 1	2.0	1.0, 2.5	1.8	0, 4.0	0.393
Day 2	1.0	0, 2.0	0.5	0, 4.0	0.353
Day 3	0.5	0.5, 1.0	0.5	0, 1.0	1.0
Day 10 follow-up	0	0, 2.0	0	0, 0.5	0.436
Day 30 follow-up	0	0, 0.5	0	0, 0	0.720
QOL VAS					
Day 10 follow-up	7.7	5.4, 10.0	6.7	2.0, 9.6	0.237
Day 30 follow-up	9.1	7.7, 10.0	8.9	0, 10.0	0.888
QOL (questionnaire)					
Day 10 follow-up	4.4	2.8, 5.0	4.3	2.4, 4.9	0.720
Day 30 follow-up	4.8	3.8, 5.0	5.0	2.8, 5.0	0.436
Owner perceived function					
Day 10 follow-up	1	1, 2	1.5	1, 2	0.442
Day 30 Follow-up	1	1, 2	1	1, 4	1.0

IQR = interquartile range. VAS = visual analog scale. QOL = quality of life.

*Based on Mann-Whitney U tests.

### Owner questionnaire

Results from questionnaire data are presented in Table 3. With the exception of the partial incisional automutilation, there were no owner reported wound healing issues noted in the patient diaries.

## Discussion

This is the first report of the use of the harmonic Osteovue blade in canine patients undergoing spinal decompression for thoracolumbar disk extrusions. Client owned dogs with spontaneous diseases such as cancer, osteoarthritis and diabetes have been increasingly incorporated into preclinical testing as models for similar human conditions as these can be more reflective of clinical outcome than data generated using induced models in laboratory animals. This has also been the case for dogs with spontaneous spinal diseases and associated surgical interventions including intervertebral disk degeneration and fibrosis development secondary to laminectomy [30, 31]. Data generated from this clinical trial provide evidence in a relevant, spontaneous animal model of IVDE that the harmonic Osteovue blade is safe and effective for the soft tissue approach to the canine thoracolumbar spine. While this study included a relatively small number of cases, it is the first of its kind to evaluate use of the HOB in a clinical setting. While previous experimental and clinical studies have demonstrated clear advantages of harmonic surgical blades over electrocautery [13–25, 32–34], the HOB design differs from previous blades and the spade like form is specifically aimed at improving soft tissue dissection from bone. The modifications are also designed to prevent blade breakage if the blade comes in contact with bone. This allows the HOB to be used to elevated soft tissues off bone as traditionally done using periosteal elevators. Furthermore, the previous blades used for harmonic tissue dissection were more likely to be damaged when bone was touched, limiting their potential application for surgical procedures conducted in proximity to bone.

Subjectively, the Osteovue blade demonstrated ease of use particularly for exposure of the affected vertebrae, requiring only the HOB and a Freer elevator for temporary retraction. There appeared to be excellent control of surface hemorrhage from subcutaneous tissue and muscle, and larger bleeding vessels could be spot coagulated effectively (such as the spinal branch of the intercostal or lumbar arteries). Due to the risk of iatrogenic damage, bleeding from the venous sinus within the spinal canal was not coagulated using the HOB or electrocautery. While sinus bleeding was subjectively rated as absent or minimal in most patients and bleeding from cancellous bone during the hemilaminectomy appeared similar among dogs, both could have affected postoperative PCV and TP.

Removal of soft tissues from and around bone was possible without visibly gauging or burning the bone surface or breaking the HOB. Importantly, the sound caused from blade vibrations was altered during contact with bone, providing a ready indication during the surgical procedure and thus avoiding potential complications such as thermal injury to the bone structure that could lead to thermal necrosis. The only observed intraoperative complication in the HOB group was base fracture of a spinous process of one of the 2 vertebra where the hemilaminectomy was performed. The fracture noted at the end of the surgery prior to wound closure and was believed to be secondary to undercutting of supporting bone during hemilaminectomy. The affected dog was very small with thin spinous process bone, which would not require a tremendous stress riser to induce a fracture if weakened. While retrospective evaluation of video recording of the surgical approach in this patient did not identify prolonged HOB contact with bone, it cannot be entirely ruled out as the cause of the injury.

Despite validated pain scales and assessment tools, evaluation of postoperative pain in dogs can be challenging. We chose the Glasgow composite pain scale for assessment of generalized signs of pain and wound pain while dogs were in the hospital [28]. Dogs in both groups demonstrated an immediate reduction of pain at 1 day postoperative compared to preoperative, likely due to reduced pain after spinal cord decompression as well as administration of effective analgesia. As expected, dogs in both groups had decreasing pain scores over time. However, dogs in the HOB group had a significantly higher improvement in pain scores (for both the questionnaire and visual analog scale) relative to their preoperative values at time of discharge and at 30 days compared to the TRAD group. This may have been secondary to the fact that dogs in this group started off with a higher pain score, which then allowed them to improve more dramatically. Similarly, neurological recovery was different between groups at time of discharge and at 10 day follow-up relative to preoperative neurologic grades with dogs in the HOB group demonstrating significantly greater improvement at both time points compared to the TRAD group. As with the pain scores, it is possible that the more severe neurological presentation in the HOB group at time of presentation resulted in greater improvement potential for those dogs.

While a variety of wound assessment scores have been described to evaluate healing of open wounds, such as pressure sores or granulating wounds [35, 36], there appear to be no validated scoring systems assessing incisional healing. The wound assessment score used in this study has not been validated nor was an attempt made to correlate visual inspection with photographs obtained. Postoperative wound healing proceeded well in both groups with 2 complications occurring. One dog in the HOB group presented with a self-inflicted partial incisional dehiscence after the dog had chewed out several skin staples on day 10 just prior to the 10 day recheck appointment. Up to that event, the incision was opposed without signs of drainage, swelling or pain according to the owner. Another dog in the HOB group had a small soft, non-painful swelling palpable near the incision diagnosed at day 30. This was thought to be a seroma although no definitive diagnosis was made through aspiration. Therefore, the post-surgical complication rates associated with both groups was very low.

While not reaching significance (possibly due to the small numbers of dogs in each group), lower blood loss occurred in the HOB group compared to the TRAD group (p = 0.063, Table 2). These data are consistent with human clinical studies that have documented a significant decrease in blood loss when using an ultrasonic surgical blade compared to electrocautery [19–25]. Excessive bleeding rarely occurs during the approach to the canine spine and is more likely encountered after access to the spinal canal through bleeding from the venous sinus, vertebral artery or intervertebral vascular branches. One weakness associated with assessment of blood loss related to the employed method, namely measuring blood collected in the suction containers; several dogs had so little bleeding that blood did not reach the container and the small amounts of blood collected within the suction tubing could not be readily assessed. More accurate methods of measuring blood loss may have altered our results.

We specifically compared the surgical approach time between groups as this was the period during HOB use and found no difference. This may be explained by the small area of surgical approach used to access the ruptured disc. To maintain a similar degree of soft tissue dissection among the two groups, dogs were only eligible if no more than 1 disc space was affected and required surgery. Evaluation of larger patients requiring more approach exposure may provide different data and document a difference in surgical time as previously described for other ultrasonic surgical blades [25].

## Conclusion

This prospective randomized clinical trial demonstrates that the HOB is a safe and effective tool for the soft tissue approach for routine spinal surgery in dogs, supporting future application to human spinal surgery. Further studies involving larger canine patients possessing multiple affected disc spaces would be required to more accurately assess its impact on intraoperative bleeding, surgical time and postoperative wound healing.

## Supporting information

S1 File Wound assessment form. Document to record various aspects of wound related factors for individual patients.
(DOCX)

S2 File Pain assessment form. Document to record various aspects of pain related factors for individual patients.
(DOCX)

S3 File Quality of life questionnaire. Document with various questions relating to the patient’s owner perceived quality of life.
(DOCX)

S1 Fig Photographs of incisions of 1 TRAD and HOB dog. Examples of incision photographs of one dog with TRAD and one with HOB approach on day of surgery and 1, 2, and 3 days postoperatively.
(JPG)

S1 Data Excel study data. Dataset of evaluated factors of all patients, including patient and surgery factors, neurologic grading, pain questionnaire and visual analog scores, incision healing scores, owner perceived neuro status and function, and quality of life factors.
(XLSX)
