# Supplementary material for: Comparison of harmonic blade versus traditional approach in canine patients undergoing spinal decompressive surgery for naturally occurring thoracolumbar disk extrusion
Source: PLoS One. 2017 Mar 2;12(3):e0172822. doi: 10.1371/journal.pone.0172822 (PMC5333832; doi:10.1371/journal.pone.0172822)
Supplement: S1 File — Document to record various aspects of wound related factors for individual patients. (DOCX) [file pone.0172822.s001.docx]

| Patient Sticker |
| --- |
|  |

Animal ID: ________

Date: ______________
 (DDMMMYY)

**Wound Assessment Form**

**Visit:** Day 0  Day 1 Day 2 Day 3 Day 10 Day 30 Unscheduled (Day__)

| Incision apposed | Yes  No |
| --- | --- |
| Minimal Moderate Severe | |
| Discharge 1 2 3 4 5  (circle one) | Yes  No |
| Swelling 1 2 3 4 5  (circle one) | Yes  No |
| Bruising 1 2 3 4 5  (circle one) | Yes  No |
| Pain on stroking over incision 1 2 3 4 5  (circle one) | Yes  No |
| Pain on palpation of surgical site 1 2 3 4 5  (circle one) | Yes  No |
| Photo (with measurement) | Yes  No |

| **Examined by:** |  | **Date:** |  |
| --- | --- | --- | --- |
| (Examining Veterinarian) |  |  | (DDMMMYY) |
| **Recorded by:** |  | **Date:** |  |
|  | **(If different than examined by)** |  | (DDMMMYY) |

**If yes to any, describe:**
