# Supplementary material for: Comparison of harmonic blade versus traditional approach in canine patients undergoing spinal decompressive surgery for naturally occurring thoracolumbar disk extrusion
Source: PLoS One. 2017 Mar 2;12(3):e0172822. doi: 10.1371/journal.pone.0172822 (PMC5333832; doi:10.1371/journal.pone.0172822)
Supplement: S2 File — Document to record various aspects of pain related factors for individual patients. (DOCX) [file pone.0172822.s002.docx]

| Patient Sticker |
| --- |
|  |

Animal ID: ________

Date: ______________
 (DDMMMYY)

**Pain Assessment Form**

**Visit:** Screening  Day 0  Day 1  Day 2  Day 3  Day 10  Day 30  Unscheduled (DAY____)


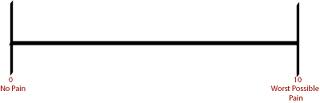


| In the section below, please circle the appropriate score in each list and sum these to give you a total score | |
| --- | --- |
| \| Look at the dog in kennel; is the dog \| \| \| \| \| --- \| --- \| --- \| --- \| \|  \|  \|  \|  \| \| Quiet \| 0 \|  \|  \| \| Crying or whimpering \| 1 \|  \|  \| \| Groaning \| 2 \|  \|  \| \| Screaming \| 3 \|  \|  \| | \| If it had a wound or painful area including abdomen, apply gentle pressure 2 inches round the site, does it? \| \| \| --- \| --- \| \|  \|  \| \| Do nothing \| 0 \| \| Look round \| 1 \| \| Flinch \| 2 \| \| Growl or guard area \| 3 \| \| Snap \| 4 \| \| Cry \| 5 \| |
| \| Overall, is the dog \|  \| \| --- \| --- \| \|  \|  \| \| Happy and content (or bouncy) \| 0 \| \| Quiet \| 1 \| \| Indifferent or non-responsive to surroundings \| 2 \| \| Nervous or anxious or fearful \| 3 \| \| Depressed or non-responsive to stimulation \| 4 \| | \| Overall, is the dog \| \|  \| \| --- \| --- \| --- \| \|  \| \|  \| \| Comfortable \| 0 \| \| \| Unsettled \| 1 \| \| \| Restless \| 2 \| \| \| Hunched or tense \| 3 \| \| \| Rigid \| 4 \| \| \|  \|  \| \| |

| **0 No discernable signs of sedation** |
| --- |
| **1 Signs of sedation but reactive to acoustic stimuli** |
| **2 Signs of sedation, no reaction to acoustic stimuli but reactive to physical examination** |
| **3 Sedated and no reaction to acoustic or physical stimuli** |

| **Examined by:** |  | **Date:** |  |
| --- | --- | --- | --- |
| (Examining Veterinarian) |  |  | (DDMMMYY) |
| **Recorded by:** |  | **Date:** |  |
|  | (If different than examined by) |  | (DDMMMYY) |
