# Supplementary material for: Comparison of harmonic blade versus traditional approach in canine patients undergoing spinal decompressive surgery for naturally occurring thoracolumbar disk extrusion
Source: PLoS One. 2017 Mar 2;12(3):e0172822. doi: 10.1371/journal.pone.0172822 (PMC5333832; doi:10.1371/journal.pone.0172822)
Supplement: S3 File — Document with various questions relating to the patient’s owner perceived quality of life. (DOCX) [file pone.0172822.s003.docx]

**INSTRUCTIONS: Please indicate your assessment by circling the number on the scale next to each question, providing your opinion on your pet’s CURRENT health status.**

**EXAMPLE:** 1 2 3 4 5

**Disagree Neutral Agree**

**HAPPINESS**

My pet wants to play 1 2 3 4 5

My pet responds to my presence 1 2 3 4 5

My pet enjoys life 1 2 3 4 5

**MENTAL STATUS**

My pet has more bad days than good days 1 2 3 4 5

My pet sleeps more, is awake less 1 2 3 4 5

My pet seems dull or depressed, not alert 1 2 3 4 5

**PAIN**

My pet is in pain 1 2 3 4 5

My pet pants frequently, even at rest 1 2 3 4 5

My pet shakes or trembles occasionally 1 2 3 4 5

**APPETITE**

My pet eats the usual amount of food 1 2 3 4 5

My pet eats treats/snacks 1 2 3 4 5

My acts nauseous or vomits 1 2 3 4 5

**WOUND**

The incision is painful to the touch 1 2 3 4 5

The incision is draining 1 2 3 4 5

The incision edges are healing well 1 2 3 4 5

**HYGIENE**

My pet can urinate voluntarily 1 2 3 4 5

My pet keeps him/herself clean 1 2 3 4 5

My pet smells like urine or has skin irritation 1 2 3 4 5

**WATER INTAKE (HYDRATION)**

My pet drinks adequately 1 2 3 4 5

My pet has normal stools 1 2 3 4 5

My pet is urinating a normal amount 1 2 3 4 5

Please circle the statement that best fits with your dog’s current neurological function:

1. Walking normally
2. Walking with some weakness and problems with coordination
3. Not walking, but movement present in affected limbs
4. Not walking and unable to move the affected limbs
5. Not walking, unable to move the affected limbs and unable to feel affected limbs

**Current quality of life**

Very Poor Excellent
