# Supplementary figures and images for: Comparison of harmonic blade versus traditional approach in canine patients undergoing spinal decompressive surgery for naturally occurring thoracolumbar disk extrusion
Source: PLoS One. 2017 Mar 2;12(3):e0172822. doi: 10.1371/journal.pone.0172822 (PMC5333832; doi:10.1371/journal.pone.0172822)

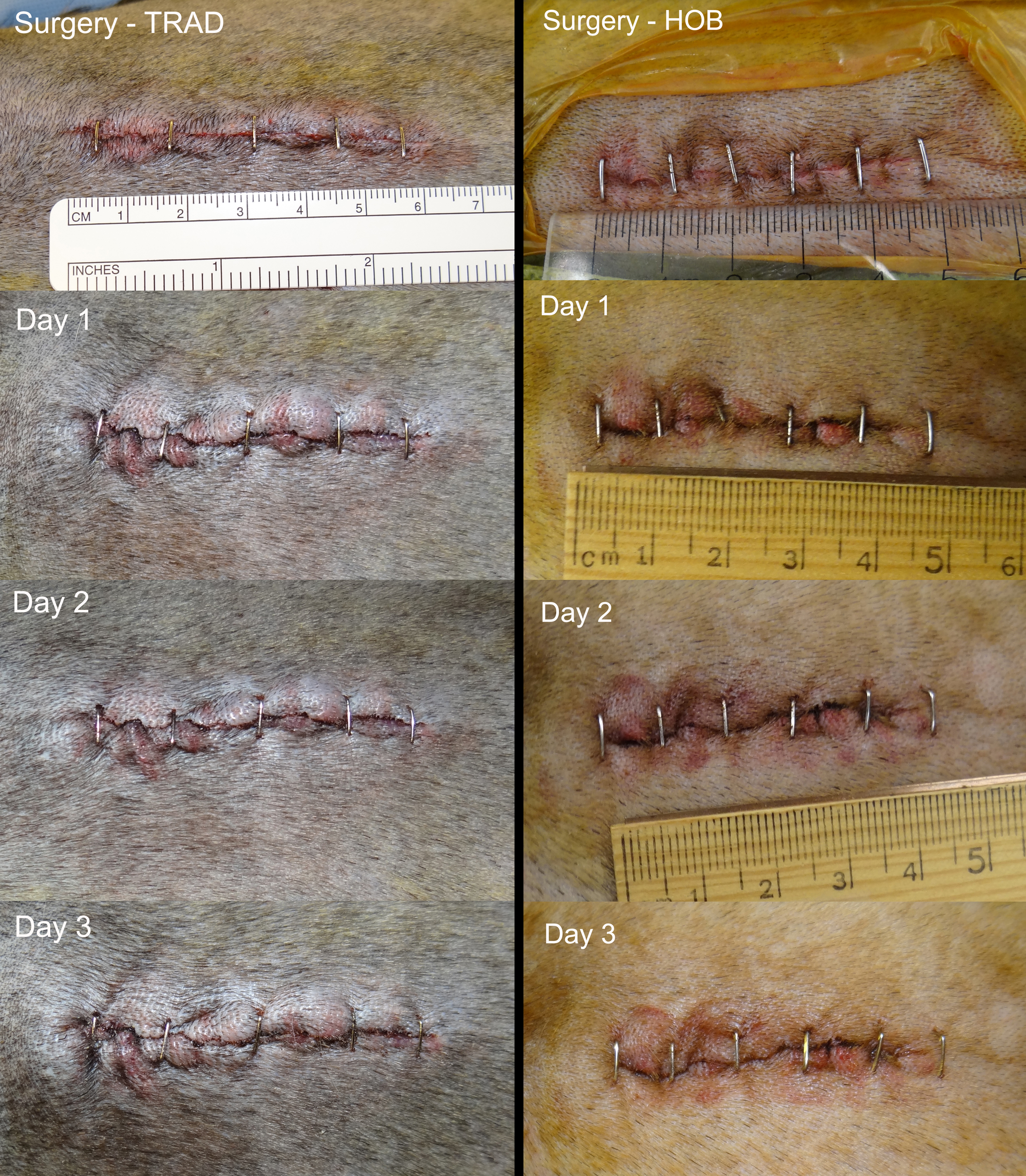

Supplement: S1 Fig — Examples of incision photographs of one dog with TRAD and one with HOB approach on day of surgery and 1, 2, and 3 days postoperatively. (JPG) [file pone.0172822.s004.jpg]
